# Supplementary material for: Effect of Yakae-Prajamduen-Jamod Traditional Thai Remedy on Cognitive Impairment in an Ovariectomized Mouse Model and Its Mechanism of Action
Source: Molecules. 2022 Jul 5;27(13):4310. doi: 10.3390/molecules27134310 (PMC9267962; doi:10.3390/molecules27134310)

Supplementary Materials

Effect of Yakae-Prajamduen-Jamod Traditional Thai Remedy on Cognitive Impairment in an Ovariectomized Mouse Model and Its Mechanism of Action

Supawadee Daodee [1], Orawan Monthakantirat [1], Ariyawan Tantipongpiradet [1,2], Juthamart Maneenet [1,3], Chantana Boonyarat [1], Charinya Khamphukdee [4], Pakakrong Kwankhao [5], Supaporn Pitiporn [5], Suresh Awale [3], Kinzo Matsumoto [6] and Yaowared Chulikhit [1,*]

[1] Division of Pharmaceutical Chemistry, Faculty of Pharmaceutical Sciences, Khon Kaen University, Khon Kaen 40002, Thailand; csupawad@kku.ac.th (S.D.); oramon@kku.ac.th (O.M.); chaboo@kku.ac.th (C.B.); juthamart_m@kkumail.com (J.M.); yaosum@kku.ac.th (Y.C.)

[2] Department of Pharmacy, University of Naples Federico II, 80131 Naples, Italy; ariyawanps@gmail.com (A.T.)

[3] Division of Natural Drug Discovery, Institute of Natural Medicine, University of Toyama, 2630 Sugitani, Toyama 930-0194, Japan; suresh@u-toyama.ac.jp (S.A.)

[4] Division of Pharmacognosy and Toxicology, Faculty of Pharmaceutical Sciences, Khon Kaen University, Khon Kaen 40002, Thailand; charkh@kku.ac.th (C.K.)

[5] Department of Pharmacy, Chao Phya Abhaibhubejhr Hospital, Ministry of Public Health, Prachinburi 25000, Thailand; pakakrong2@gmail.com (P.K.); spitiporn@yahoo.com (S.P.)

[6] Graduate School of Pharmaceutical Sciences, Daiichi University of Pharmacy, Fukuoka 815-8511, Japan; k-matsumoto@daiichi-cps.ac.jp (K.M.)

* Correspondence: yaosum@kku.ac.th; Tel.: +66-81-380 2357

1. Statistical Analysis Effect of YPJ formulation on OVX-Induced Cognitive Dificit Behavioral Using Y-maze test.

Table S1. One-way analysis of variance (ANOVA) test of Y-maze test.

Group comparison	Statistical Analysis	
	P	*F* ($DF_{between\ group}$, $DF_{residual}$)
Sham vs. OVX + vehicle group (t-Test)	0.002	
ANOVA followed by Tukey's post hoc test		
All group	<0.001	
OVX + vehicle group vs. OVX + Eestradiol	<0.001	$F(3,44) = 6.975$
OVX + vehicle group vs. OVX + YPJ100	0.477	
OVX + vehicle group vs. OVX + YPJ500	0.044	
OVX + YPJ 100 vs. OVX + YPJ 500	0.581	

2. Statistical Analysis Effect of of YPJ formulation on OVX-Induced Cognitive Dificit Behavioral Using Novel Object Recognition Test (NORT).

Table S2. One-way analysis of variance (ANOVA) test of novel object recognition test (NORT).

Group comparison	Statistical Analysis	
	P	*F* ($DF_{between\ group}$, $DF_{residual}$)
Sham vs. OVX + vehicle group (t-Test)	0.002	
ANOVA followed by Tukey's post hoc test		
All group	<0.001	
OVX + vehicle group vs. OVX + Eestradiol	<0.001	$F(3,44) = 6.975$
OVX + vehicle group vs. OVX + YPJ100	0.324	
OVX + vehicle group vs. OVX + YPJ500	<0.001	
OVX + YPJ 100 vs. OVX + YPJ 500	0.035	

3. Statistical Analysis Effect of YPJ formulation on OVX-Induced Cognitive Dificit Behavioral Using Morris Water Maze Test (MWMT).

Table S3.1. One-way analysis of variance (ANOVA) test of MWMT on the training test (Day 5).

Group comparison	Statistical Analysis	
	P	*F* ($DF_{between\ group}$, $DF_{residual}$)
Sham vs. OVX + vehicle group (t-Test)	<0.001	
ANOVA followed by Tukey's post hoc test		
All group	<0.001	
OVX + vehicle group vs. OVX + Eestradiol	<0.05	$F(3,44) = 17.548$
OVX + vehicle group vs. OVX + YPJ100	<0.05	
OVX + vehicle group vs. OVX + YPJ500	<0.001	
OVX + YPJ 100 vs. OVX + YPJ 500	<0.001	

Table S3.2. One-way analysis of variance (ANOVA) test of MWMT on the probe test.

Group comparison	Statistical Analysis	
	P	*F* ($DF_{between\ group}$, $DF_{residual}$)
Sham vs. OVX + vehicle group (t-Test)	<0.001	
ANOVA followed by Tukey's post hoc test		$F(3,44) = 12.168$
All group	<0.001	
OVX + vehicle group vs. OVX + Eestradiol	<0.001	
OVX + vehicle group vs. OVX + YPJ100	<0.05	
OVX + vehicle group vs. OVX + YPJ500	<0.001	
OVX + YPJ 100 vs. OVX + YPJ 500	0.433	

4. Statistical Analysis Effect of YPJ formulation on Serum CORT Level

Table S4. One-way analysis of variance (ANOVA) test of serum CORT level.

Group comparison	Statistical Analysis	
	P	*F* ($DF_{between\ group}$, $DF_{residual}$)
Sham vs. OVX + vehicle group (t-Test)	<0.001	
ANOVA followed by Tukey's post hoc test		$F(3,16) = 88.315$
All group	<0.001	
OVX + vehicle group vs. OVX + Eestradiol	<0.001	
OVX + vehicle group vs. OVX + YPJ100	<0.05	
OVX + vehicle group vs. OVX + YPJ500	<0.001	
OVX + YPJ 100 vs. OVX + YPJ 500	<0.001	

5. Statistical Analysis Effect of YPJ formulation on OVX-Changes Antioxidant enzymes in the Frontal cortex and Hippocampus.

Table S5.1. One-way anaysis of variance (ANOVA) test of OVX-Changed CAT activity in frontal cortex and hippocampus.

Group comparison	ANOVA followed by Tukey's post hoc test	
Frontal cortex	*P*	*F* ($DF_{between\ group}$, $DF_{residual}$)
Sham vs. OVX + vehicle group	0.015	
ANOVA followed by Tukey's post hoc test		$F(3,16) = 10.031$
All group	<0.001	
OVX+ vehicle group vs. OVX + E_2	<0.001	
OVX+ vehicle group vs. OVX + YPJ 100	0.360	
OVX+ vehicle group vs. OVX + YPJ 500	0.006	
OVX + YPJ 100 vs. OVX + YPJ 500	0.146	
Hippocampus	*P*	*F* ($DF_{between\ group}$, $DF_{residual}$)
Sham vs. OVX + vehicle group	<0.001	
ANOVA followed by Tukey's post hoc test		$F(3,16) = 12.089$
All group	<0.001	
OVX + vehicle group vs. OVX + E_2	0.023	
OVX + vehicle group vs. OVX + YPJ 100	0.374	

OVX + vehicle group vs. OVX + YPJ 500	<0.001	
OVX + YPJ 100 vs. OVX + YPJ 500	0.014	

Table S5.2. One-way analysis of variance (ANOVA) test of OVX-Changed SOD activity in frontal cortex and hippocampus.

Group comparison	ANOVA followed by Tukey's post hoc test	
Frontal cortex	*P*	*F* ($DF_{between\ group}$, $DF_{residual}$)
Sham vs. OVX + vehicle group	0.002	
ANOVA followed by Tukey's post hoc test		
All group	<0.001	*F*(3,16) = 10.035
OVX + vehicle group vs. OVX + E_2	0.006	
OVX + vehicle group vs. OVX + YPJ 100	1.000	
OVX + vehicle group vs. OVX + YPJ 500	0.009	
OVX + YPJ 100 vs. OVX + YPJ 500	0.008	
Hippocampus	*P*	*F* ($DF_{between\ group}$, $DF_{residual}$)
Sham vs. OVX + vehicle group	0.001	
ANOVA followed by Tukey's post hoc test		
All group	<0.001	*F*(3,16) = 17.833
Sham vs. OVX + vehicle group	0.003	
OVX + vehicle group vs. OVX + E_2	<0.05	
OVX + vehicle group vs. OVX + YPJ 100	0.374	
OVX + vehicle group vs. OVX + YPJ 500	0.004	
OVX + YPJ 100 vs. OVX + YPJ 500	<0.001	

6. Statistical Analysis Effect of YPJ formulation on OVX-Changes Proinflammatory Cytokines in the Frontal cortex and Hippocampus.

Table S6.1. One-way analysis of variance (ANOVA) test of OVX-Changed *IL-1β* expression in frontal cortex and hippocampus.

Group comparison	ANOVA followed by Tukey's post hoc test	
Frontal cortex	*P*	*F* ($DF_{between\ group}$, $DF_{residual}$)
All group	0.019	*F*(3,16) = 4.407
Sham vs. OVX + vehicle group	<0.001	
OVX + vehicle group vs. OVX + E_2	0.022	
OVX + vehicle group vs. OVX + YPJ 100	0.4034	
OVX + vehicle group vs. OVX + YPJ 500	0.047	
OVX + YPJ 100 vs. OVX + YPJ 500	0.549	
Hippocampus	*P*	*F* ($DF_{between\ group}$, $DF_{residual}$)
All group	0.03	*F*(3,16) = 7.059
Sham vs. OVX + vehicle group	<0.05	
OVX + vehicle group vs. OVX + E_2	0.004	
OVX + vehicle group vs. OVX + YPJ 100	0.555	

OVX + vehicle group vs. OVX + YPJ 500	0.017	
OVX + YPJ 100 vs. OVX + YPJ 500	0.202	

Table S6.2 One-way analysis of variance (ANOVA) test of OVX-Changed *IL-6* expression in frontal cortex and hippocampus.

Group comparison	ANOVA followed by Tukey's post hoc test	
Frontal cortex	*P*	*F* ($DF_{between\ group}$, $DF_{residual}$)
All group	<0.001	$F(3,16) = 32.497$
Sham vs. OVX + vehicle group	<0.001	
OVX+ vehicle group vs. OVX + E_2	<0.001	
OVX + vehicle group vs. OVX + YPJ 100	<0.001	
OVX + vehicle group vs. OVX + YPJ 500	<0.001	
OVX + YPJ 100 vs. OVX + YPJ 500	0.341	
Hippocampus	*P*	*F* ($DF_{between\ group}$, $DF_{residual}$)
All group	<0.001	$F(3,16) = 21.856$
Sham vs. OVX + vehicle group	<0.001	
OVX + vehicle group vs. OVX + E_2	<0.001	
OVX + vehicle group vs. OVX + YPJ 100	0.398	
OVX + vehicle group vs. OVX + YPJ 500	0.001	
OVX + YPJ 100 vs. OVX + YPJ 500	0.024	

Table S6.3. One-way analysis of variance (ANOVA) test of OVX-Changed *TNF-α* expression in frontal cortex and hippocampus.

Group comparison	ANOVA followed by Tukey's post hoc test	
Frontal cortex	*P*	*F* ($DF_{between\ group}$, $DF_{residual}$)
All group	<0.001	$F(3,16) = 166.516$
Sham vs. OVX + vehicle group	<0.001	
OVX + vehicle group vs. OVX + E_2	<0.001	
OVX + vehicle group vs. OVX + YPJ 100	0.776	
OVX + vehicle group vs. OVX + YPJ 500	<0.001	
OVX + YPJ 100 vs. OVX + YPJ 500	<0.001	
Hippocampus	*P*	*F* ($DF_{between\ group}$, $DF_{residual}$)
All group	<0.001	$F(3,16) = 9.53$
Sham vs. OVX + vehicle group	<0.05	
OVX + vehicle group vs. OVX + E_2	<0.001	
OVX + vehicle group vs. OVX + YPJ 100	0.26	
OVX + vehicle group vs. OVX + YPJ 500	0.043	
OVX + YPJ 100 vs. OVX + YPJ 500	0.74	

Table S7. Validation results of the analytical method for determination of gallic acid, myricetin, quercetin, luteolin, genistein and coumestrol in YPJ extract.

Parameter		Compounds					
		Gallic acid (1)	Myricetin (2)	Quercetin (3)	Luteolin (4)	Genistein (5)	Coumestrol (6)
Linearity	Range (µg/mL)	1–10	1–10	1–10	1–10	1–10	1–10
	Coefficient determination (R^2)	0.9998 ± 0.00011	0.9963 ± 0.00145	0.9996 ± 0.00008	0.9969 ± 0.00049	0.9977 ± 0.00050	0.9995 ± 0.00005
LOD (µg/mL)		1 (S/N ~ 3.48 ± 0.19)	0.5 (S/N ~ 2.97 ± 0.17)	0.5 (S/N ~ 5.25 ± 0.40)	1 (S/N ~ 4.75 ± 0.36)	0.1 (S/N ~ 3.62 ± 0.14)	0.5 (S/N ~ 2.71 ± 0.12)
LOQ (µg/mL		3 (S/N ~ 10.34 ± 0.54)	3 (S/N ~ 11.86 ± 1.59)	1 (S/N ~ 12.42 ± 0.59)	3 (S/N ~ 11.28 ± 0.41)	0.25 (S/N ~ 10.12 ± 0.48)	2 (S/N ~ 14.63 ± 1.72)
Precision (% RSD)	Within day	0.107–0.446	0.121–1.950	0.088–0.427	0.121–0.707	0.225–1.577	0.088–0.353
	Between day	0.308–0.675	0.785–2.799	0.457–2.018	0.252–2.469	0.342–2.669	0.593–1.181
Accuracy (% Recovery)	Low (µg/mL)	102.59 ± 0.63	123.85 ± 3.21	109.44 ± 0.25	123.41 ± 1.23	125.48 ± 0.62	107.70 ± 0.85
	Midium (µg/mL)	99.77 ± 0.37	98.97 ± 2.09	98.59 ± 0.30	100.62 ± 1.12	97.45 ± 0.28	99.21 ± 0.24
	High (µg/mL)	100.85 ± 1.30	103.32 ± 1.91	100.45 ± 0.72	101.41 ± 1.25	102.10 ± 0.61	100.76 ± 0.25

Figure S1. HPLC chromatograms of six standards solution gallic acid (1), myricetin (2), quercetin (3), luteolin (4), genistein (5) and coumestrol (6).

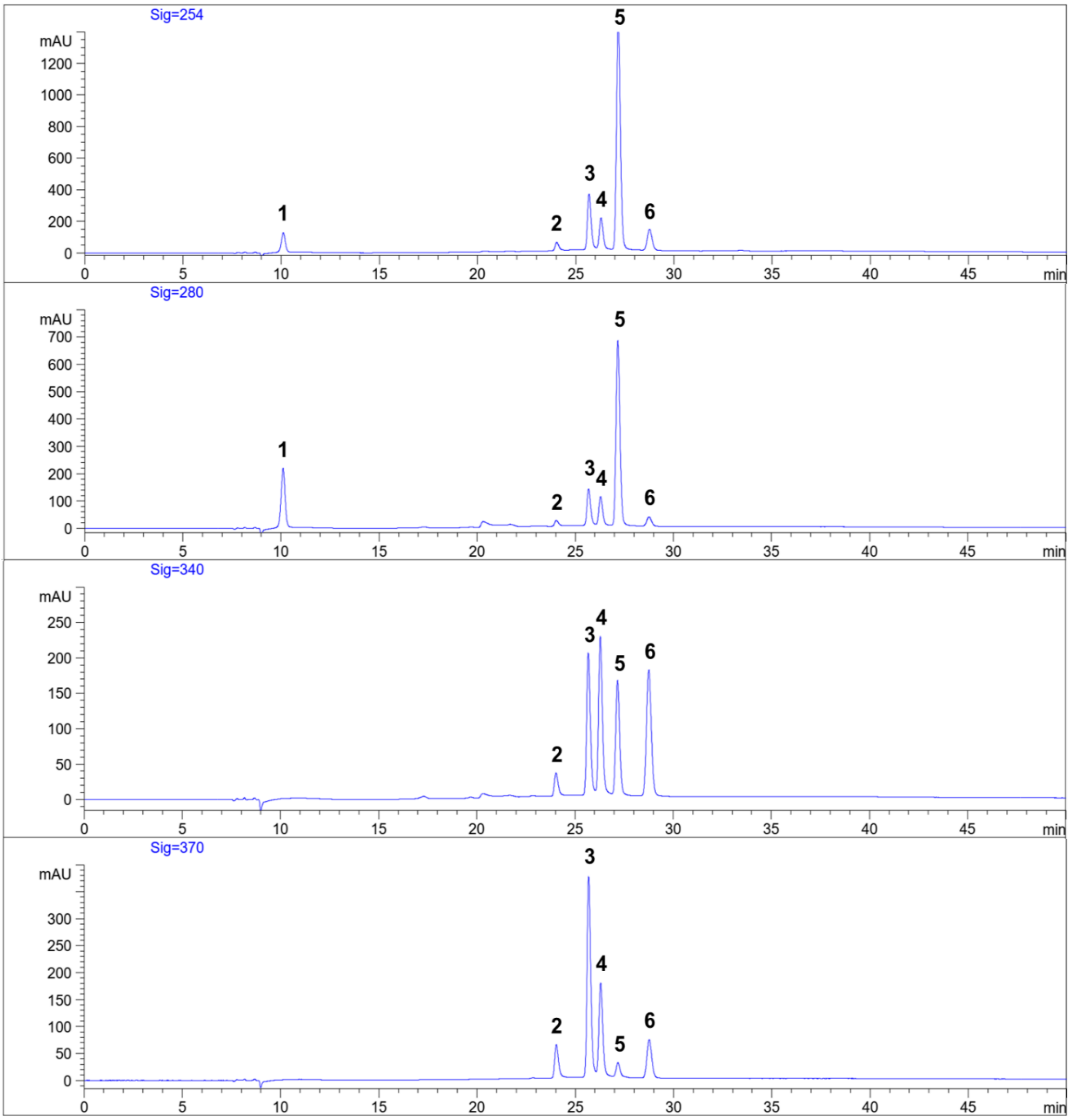

Figure S2. HPLC chromatograms of six standards solution gallic acid (1), myricetin (2), quercetin (3), luteolin (4), genistein (5) and coumestrol (6) in the YPJ extract.

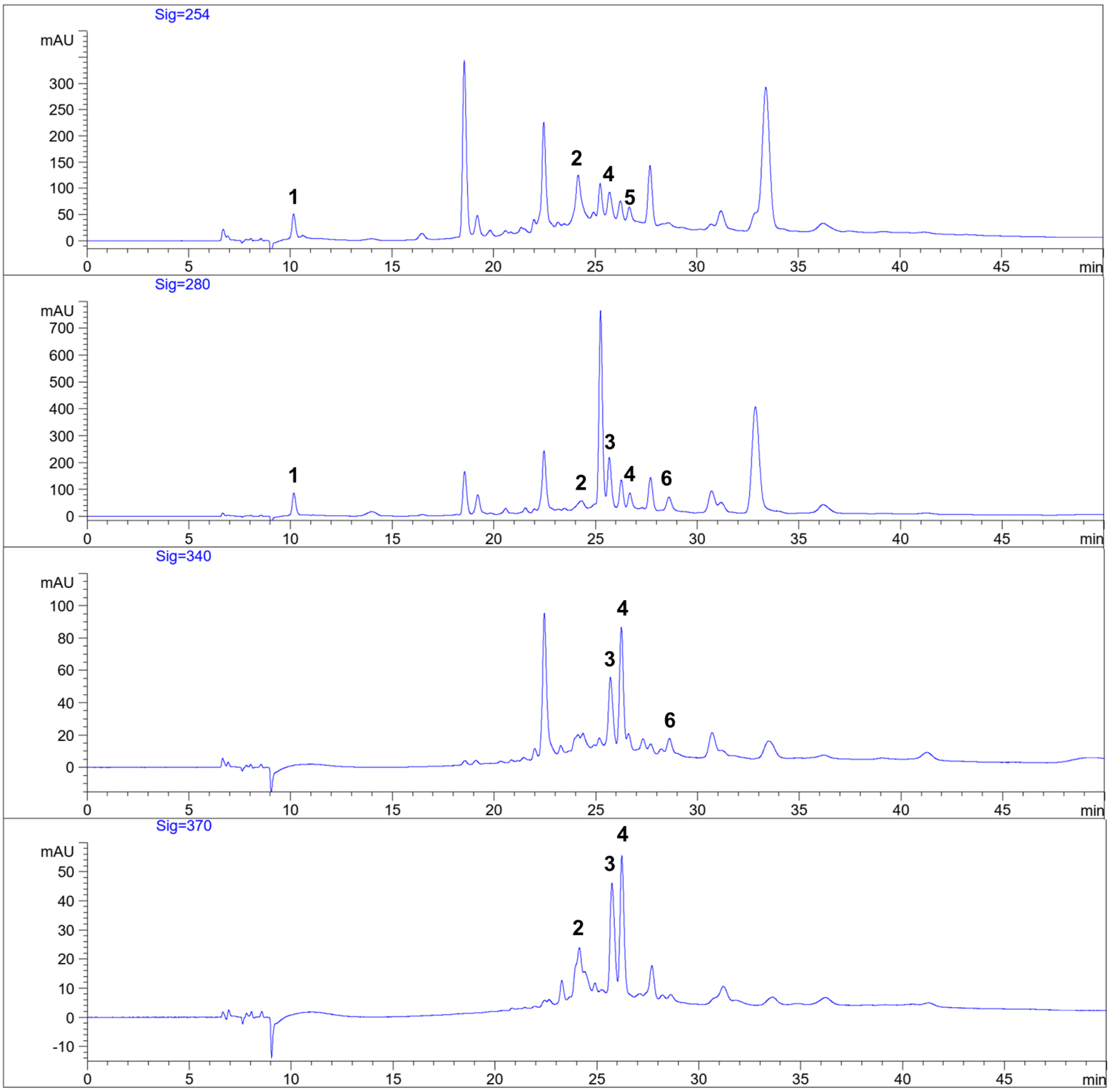

Supplement: Supplementary file 1 [file molecules-27-04310-s001.zip › molecules-1766620-supplementary.pdf]
